# Characterization of *Serratia marcescens* (OK482790)’ prodigiosin along with in vitro and in silico validation for its medicinal bioactivities

**DOI:** 10.1186/s12866-024-03634-5

**Published:** 2024-11-25

**Authors:** Marwa A. Hamada, Eslam T. Mohamed

**Affiliations:** Botany and Microbiology Department, Faculty of Science, Helwan University, Helwan, Egypt

**Keywords:** Bioactive pigment, Biological activities, Fourier-transform infrared spectroscopy, Mass Spectroscopy, Molecular docking, Rhizosphere, Thin-layer chromatography

## Abstract

**Background:**

Microbial prodigiosin pigment has been proposed as a promising biomolecule having an antibacterial, immunosuppressive, antimalarial, antineoplastic, and anticancer activities. The good outcome originates from getting natural pigment, which has many medical applications.

**Results:**

In this investigation, prodigiosin (PG) was extracted, characterized by UV-visible spectroscopy, thin-layer chromatography, mass spectroscopy, Fourier-transform infrared spectroscopy, and tested in various medical applications as an antibacterial, antioxidant, antibiofilm, anticancer, and wound healing agent at different concentrations. Antibacterial activity of PG pigment was shown against both Gram-positive and Gram-negative bacterial strains. *Enterococcus faecalis* was the most severely impacted, with minimum inhibitory value of 3.9 µg/mL. The formed biofilm by *Pseudomonas aeruginosa* was suppressed by 58–2.50% at prodigiosin doses ranging from 1000 to 31.25 µg/mL, respectively. The half-maximal inhibitory concentration (IC_50_) of 2,2’-azino-bis (3-ethylbenzothiazoline-6-sulfonic acid (ABTS) free radical was 74.18 ± 23.77 µg/mL. At 100 µg/mL concentration, OK482790 prodigiosin had no harmful effect on normal skin cells and exhibited mild wound healing properties. Additionally, molecular docking simulations confirmed the prodigiosin’s interactions with target proteins, including epidermal growth factor receptor tyrosine kinase (EGFR-TK, PDB ID: 1M17), peptide deformylase from *E. faecalis* (PDB ID: 2OS1), acidic fibroblast growth factor (FGF-1, PDB ID: 3K1X), PA14_16140 protein from *P. aeruginosa* (PDB ID: 8Q8O), and human peroxiredoxin 5 (PDB ID: 1HD2) for explaining the anticancer, antibacterial, wound healing, antibiofilm, and antioxidant activities, respectively. Prodigiosin had favorable binding affinities and putative modes of action across various therapeutic domains.

**Conclusion:**

This study pioneers the use of prodigiosin as a natural alternative to synthetic medicine since it fights germs, heals wounds, is antioxidant, and reduces biofilm formation.

**Clinical trial number:**

Not applicable.

## Background

The pigments in an organism are responsible for its coloration. Pigments find widespread application as colorants, additives, and antioxidants in several industries, including agriculture, clothing, medicine, and cosmetics. Both natural and synthetic pigments are the most common kinds [1]. Because some synthetic pigments are toxic and cause allergies, mutations, cancer, and other problems, people from all over the world are becoming more interested in natural pigments that are healthy and safe [2]. Pigments found in nature are often derived from either plants or microbes [3]. Microbial pigments offer a number of benefits over plant pigments, including higher yields, lower costs, and ease of handling after production. Also, there is serious concern that large-scale production of plants to get their pigments could harm the ecosystem in many ways, such as by causing deforestation and reducing the number of species that live there [4]. Pigments such as carotenoids, melanins, flavonoids, and phenazines are secondary metabolites that have been found to be produced by microorganisms [5]. Prodigiosin (PG) is an alkaloid secondary metabolite can be produced by a wide variety of microbes, such as *Serratia marcescens*, *S. rubidaea*, *Pseudomonas magneslorubra*, *Pseudovibrio denitrificans*, *Pseudoalteromonas rubra*, *Vibrio psychroerythrous*, *V. gazogenes*, *Streptomyces lividans*, and *Nocardia* spp [6]. *Serratia* species are Gram-negative, rod-shaped bacteria, facultatively anaerobic bacteria that are found everywhere and belong to the family Enterobacteriaceae [7]. They are found in many places, including the air, water, soil, plants, fruits, and animals [3]. Their ability to secrete a wide range of bioactive compounds, including surfactant serrawettin, extracellular enzymes (chitinases, nucleases, lipases, and proteases), and antifungals, carbapenems, and prodigiosins, renders them beneficial in agriculture biocontrol [8]. So far, there are 18 known *Serratia* species. Four of them, *S. marcescens*, *S. nematodiphila*, *S. plymuthica*, and *S. rubidaea*, have been described and named based on their ability to make prodigiosin [7]. Prodigiosin [9], 2,3-butanediol [10], serratamolide [11], althiomycin [12], acetoin [13], and a variety of other valuable compounds are all manufactured by *S. marcescens*. The red linear tripyrrole pigment prodigiosin (PG) has attracted a significant amount of focus because of its immunosuppressant, antibacterial, antifungal, antiprotozoal, antimalarial, antibiofilm, and antiviral properties [14, 15]. PG exhibits antimicrobial activity against a wide range of bacteria and fungi, which are the main culprits behind a number of illnesses and food spoilage. This data opens the door for PG to be used as a promising antibacterial agent for food packaging or wound healing applications [16]. Moreover, prodigiosin has been observed to prevent the production of biofilms by pathogenic bacteria, including *Salmonella typhimurium*, *E. coli*, *Pseudomonas aeruginosa*, *Enterococcus faecalis*, methicillin-resistant *Staphylococcus aureus* (MRSA), and *Staphylococcus aureus* [17]. Prodigiosin has piqued the interest of numerous sectors, including academia, the nutraceutical, pharmaceutical, cosmetic, and others, because of its amazing potential [6]. Extracellular and cell-associated vesicles, in addition to intracellular granules, are common locations for this pigment. Its chemical structure is made up of three pyrrolic rings, which give it an intense red color that is very similar to blood [4]. Many tri-pyrrole derivatives have shown potential as pro-apoptotic agents in the fight against cancer and drug-resistant cells [18]. Extensive analytical techniques are utilized to assess prodigiosin and its derivatives both qualitatively and quantitatively. These techniques include UV-visible spectroscopy, nuclear magnetic resonance (NMR), high-performance liquid chromatography (HPLC), GC/MS, TLC, and FTIR [19].

Our attention was drawn to the red and shiny colonies of *Serratia marcescens* (OK482790) found in the Lupin rhizosphere. In the current research, the red pigment was extracted, analyzed using various chemical methods such as UV-visible spectroscopy, GC/MS, TLC, and FTIR, partially purified, and assessed in some medical applications such as antibacterial, antioxidant, antibiofilm, anticancer, and wound healing agent.

## Methods

### Bacterial strains

A red pigmented strain of *Serratia marcescens* (OK482790) was isolated from the Lupin rhizosphere, and this strain was used as the source of prodigiosin pigment production [20]. For antibacterial activity, various reference bacterial strains were used, as follows: *Enterococcus faecalis* ATCC 29212, *Escherichia coli* ATCC 8739, *Staphylococcus aureus* ATCC 6538, *Clostridium perfringens* ATCC 13124, *Salmonella typhimurium* ATCC 14028, *Shigella sonnei* ATCC 29930, *Listeria monocytogenes* ATCC 7644, and *Pseudomonas aeruginosa* ATCC 27853. For the antibiofilm assay, *Pseudomonas aeruginosa* ATCC 27853 was used. Every month, the strains were cultivated on tryptic soy agar (TSA) slants and kept at 4ºC until they were needed again.

## Prodigiosin extraction

Pigment extraction was performed in accordance with Gohel et al. [6], with minor modifications as follows: First, the OK482790 strain was previously cultured overnight on a TSA slant and incubated at 28°C, then the cells were spread on the surface of nutrient agar (NA) plates and incubated at 28°C for 24 h. Following that, the pigmented cultures were scraped off the surface of NA plates. The pigment was extracted from the cells by using ethanol as a solvent and sonicating the cells for 5 min at a pulsed power of 40% and a pulsar rate of 30% by using an ultrasonic homogenizer (BioLogics Inc., USA), then the mixture was kept for a period of 24 h in the refrigerator. The residual cells were removed by centrifugation (Sigma, Germany) at 4°C and 10.000 rpm for 10 min. The crude extract was evaporated in the desiccator to dryness. After that, the pigment was sterilized using a 0.45 µL pore-sized Millipore cellulose nitrate membrane filter (CHM, Spain) after determining its amount on a dry weight basis.

## Prodigiosin purification

The red pigmented crude extract was partially purified on ready-made preparative TLC silica gel sheets 20 × 20 cm (Merck, Germany) using 9:1 chloroform/methanol as the solvent system. The sample was carefully placed on the plate 1 cm from the bottom and then placed in each of the developing chambers that contained the appropriate solvent ratios. Once the solvent font reached the indicated line, the plates were carefully removed and allowed to air-dry. Following that, the pigment of interest was scraped, eluted from silica with the same solvent system, air dried, and dissolved in ethanol for additional study.

## Prodigiosin characterization

### Prodigiosin preliminary identification and UV–vis spectral analysis

For preliminary identification of prodigiosin, the pigment color was tested under both acidic and alkaline conditions. So, two equal parts of the pigment were prepared. After that, drops of strong hydrochloric acid and sodium hydroxide solution were used to acidify and alkalinize the two parts, respectively. Using a spectrophotometer, both acidic and basic solutions of prodigiosin pigment were evaluated for maximal UV-vis absorbance between 200 and 800 nm. As a control, pure ethanol was used [21].

### Thin-layer chromatography (TLC)

The procedure was carried out using a solvent system that consisted of a chloroform and methanol (9:1) mixture and TLC silica gel plates (Merck, Germany), and retardation factor (R_f_) was determined as mentioned in Poddar et al. [22].

## GC/MS analysis

The molecular weight of the red prodigiosin pigment was obtained by a gas chromatography/mass spectrometer (Thermo Scientific, Austin, TX, USA) with a direct capillary column TG–5MS (30 m x 0.25 mm x 0.25 μm film thickness). The temperature of the column oven was kept at 60°C to start, then raised to 250°C with a 2-min hold, and finally raised to 300°C with a 30°C/min. The injector’s temperature was maintained at 270°C. As a carrier gas, helium was employed continuously, delivering 1 mL/min. The solvent delay was 4 min, and diluted samples of 1 µL were injected automatically using an Autosampler AS3000 linked with GC in split mode. Full scan mode was used to gather electron ionization (EI) mass spectra from m/z 50–650 across 70 eV ionization voltages. Temperatures of 200°C and 280°C were used for the ion source and transfer line, respectively. The mass spectra of elements were contrasted with those in the WILEY 09 and NIST 14 databases in order to identify them.

## FTIR spectroscopy

In the central lab of the Faculty of Science, Helwan University, Cairo, Egypt, the partially purified red pigment was analyzed using Fourier transform infrared spectroscopy. The distinct functional groups were generated by mixing the dehydrated pigment with KBr, and the spectral range was between 450 and 4000 cm^− 1^ using a PerkinElmer L1600400 FTIR spectrometer, UK [21].

### Application of prodigiosin pigment

#### Antibacterial activity

The test was based on the broth microdilution assay that had been previously described by Balouiri et al. [23] and CLSI [24], with the following minor modifications: 500 µg of red prodigiosin pigment was dissolved in 1 mL of ethanol. Onto the initial microtiter plate column, 400 µL of stock solution was added; subsequently, 200 µL of sterile tryptic soy broth (TSB) was added in each well (from 2 to 12). A two-fold dilution was accomplished by shifting 200 µL from the first well to the ninth. Each well in a single row, with the exception of the last one used as a blank, was supplemented with 50 µL of each reference strain containing 1 × 10^7^ CFU/mL. Then, all microtiter plates were kept at 37^o^C for 24 h. ChroMate 4300, USA Elisa Reader was used to read the results at 630 nm. Chloramphenicol (1 mg/mL) and ciprofloxacin (1 mg/mL) antibiotics served as the control group.

### Antioxidant activity

The antioxidant activity was detected by following the protocol of Arnao et al. [25], with minor modifications to be carried out in microplates. Briefly, in a 50-mL volumetric flask, 192 mg of 2,2’-azino-bis (3-ethylbenzothiazoline-6-sulfonic acid) (ABTS) were dissolved in distilled water. After solution preparation, 1 mL was introduced to 17 µL of 140 mM potassium persulfate, and the mixture was allowed to sit in darkness for 24 h. Then, to get the final ABTS dilution for the assay, 1 mL of the reaction mixture was diluted with 50 mL of methanol. In a 96-well plate (*n* = 6), 10 µL of prodigiosin pigment at final concentrations of 9.357, 18.75, 37.5, 75, and 150 µg/mL in methanol was combined with 190 µL of the newly made ABTS reagent. The reaction was incubated for 30 min in complete darkness. The ABTS color intensity was measured at 734 nm after the incubation time had ended. Data are represented as means ± SD according to the following equation: 𝑝𝑒𝑟𝑐𝑒𝑛𝑡𝑎𝑔𝑒 𝑖𝑛ℎ𝑖𝑏𝑖𝑡𝑖𝑜𝑛 = (𝐴𝑣𝑒𝑟𝑎𝑔𝑒 𝑎𝑏𝑠𝑜𝑟𝑏𝑎𝑛𝑐𝑒 𝑜𝑓 𝑏𝑙𝑎𝑛𝑘−𝑎𝑣𝑒𝑟𝑎𝑔𝑒 𝑎𝑏𝑠𝑜𝑟𝑏𝑎𝑛𝑐𝑒 𝑜𝑓 𝑡ℎ𝑒 𝑡𝑒𝑠𝑡/𝐴𝑣𝑒𝑟𝑎𝑔𝑒 𝑎𝑏𝑠𝑜𝑟𝑏𝑎𝑛𝑐𝑒 𝑜𝑓 𝑏𝑙𝑎𝑛𝑘) ∗ 100. The FluoStar Omega microplate reader was used to record the results. Trolox was prepared at concentrations of 2.50, 5, 6.25, 7.5, and 8.75 µg/mL as a standard antioxidant. Data was analyzed using Microsoft Excel^®^, and GraphPad Prism 6^®^ was used to calculate the IC_50_ value. The concentrations were converted to their logarithmic values, and a non-linear inhibitor regression equation (log (inhibitor) vs. normalized response to variable slope equation) was selected.

### Antibiofilm activity

The antibiofilm mechanism of prodigiosin against *Pseudomonas aeruginosa* ATCC 27853 was determined using the crystal violet quantitative assay, as published by Hamada et al. [26], with a few modifications: On a polystyrene plate with ninety-six wells, 50 µL of newly prepared *P. aeruginosa* suspension containing approximately 1 × 10^7^ CFU/mL^− 1^ (OD ~ 0.1) was applied to different concentrations of prodigiosin pigment (1000, 500, 250, 125, 62.5, 31.25, and 0 µg/mL^− 1^). After that, it was statically incubated at 37ºC for 24 h. Following the incubation period, a sterile 1X phosphate buffer solution (PBS, pH 7.3) was used to wash the wells twice in order to eradicate the bacterial planktonic cells. The biofilm was air-dried to fix it, and then it was dyed for 10 min with a 1% crystal violet solution. The last step was to add pure ethanol to each well and then use a microplate reader (ChroMate 4300, USA) to measure the absorbance at 630 nm. The outcome was expressed as a percentage of the suppression of biofilm formation using Microsoft Excel^®^ [27]. The statistical analyses were conducted using a one-way ANOVA with the SPSS statistics software version 22, and post-hoc tests (Duncan) were employed to check the significance (*p* < 0.05) between different treatments.

### Cytotoxicity and anticancer activity

The cytotoxicity of prodigiosin pigment was tested on human skin fibroblasts (HSF), which are considered to be normal skin cell lines. But the anticancer activity of the pigment was determined by using human epidermoid skin carcinoma (skin/epidermis) (A-431), and 5-fluorouracil was used as a positive control since it is one of the methods of treating skin cancer [28]. Each of the cell lines was provided by Nawah Scientific Inc. (Mokatam, Cairo, Egypt). The cells were grown in Dulbecco^’^s modified Eagle medium (DMEM) with an additional 10% heat-inactivated fetal bovine serum, 100 units/mL of penicillin, and 100 mg/mL of streptomycin in a humidified, 5% (v/v) CO_2_ environment at 37°C. The viability of the cells was evaluated using the SRB protocol as follows: Volumes of 100 µL of cell suspension (5 × 10^3^ cells) were placed in 96-well plates and left to incubate in a medium for a night. Another portion of 100 µL of medium containing prodigiosin at different concentrations was added to the cells. Following 72 h of drug treatment, the cells were incubated at 4°C for 1 h after 150 µL of 10% trichloroacetic acid (TCA) was added to the medium to fix them. The cells were rinsed five times with distilled water after the TCA solution was withdrawn. Portions of 70 µL of a sulforhodamine B (SRB) solution containing 0.4% w/v were introduced and left to incubate in a dark area at room temperature for 10 min. After three washes with 1% acetic acid, the plates were allowed to air-dry for the night. After that, a BMG LABTECH^®^-FluoStar Omega microplate reader (Ortenberg, Germany) was employed to measure the absorbance at 540 nm after adding 150 µL of TRIS (10 mM) to dissolve the protein-bound SRB dye [29].

### Scratch wound assay

Human skin fibroblast (HSF) cells used in this study were obtained from Nawah Scientific Inc. in Mokatam, Cairo, Egypt. For the scratch wound assay, cells were cultured overnight in 5% FBS-DMEM at 37°C and 5% CO_2_. After that, they were seeded at a density of 2 × 10^5^ cells per well onto a coated 12-well plate. The media used for cell maintenance included 100 mg/mL of streptomycin, 100 units/mL of penicillin, and 10% heat-inactivated fetal bovine serum. The incubator was humidified and contained 5% (v/v) CO_2_. The following day, the confluent monolayer was scratched horizontally. After that, the plate was washed with PBS. The control wells were filled with new medium, and the drug wells were treated with new media that included prodigiosin (100 µg/mL). An inverted microscope was used for taking images at regular intervals. During the incubation period, the plate was kept at 37°C with 5% CO_2_. To analyze the obtained images and determine the wound’s width, Version 3.7 of the MII Image View software was used, and the results were displayed as mean ± standard deviation using Microsoft Excel^®^. Migration rate (R_m_) was calculated by the following formula: R_m_ = W_i_ × W_f_ / t, where R_m_ is the rate of cell migration, W_i_ is the initial wound width, W_f_ is the final wound width, and t is the duration of migration (in hours) [30, 31].

### Molecular docking simulations of prodigiosin against various protein targets

The biological significance of the compound prodigiosin was investigated through molecular docking simulations targeting specific proteins for distinct activities. The protein structures were obtained from the Protein Data Bank (PDB), and the structure of prodigiosin was obtained from the PubChem database (PubChem ID: 135408511) [32]. Epidermal growth factor receptor tyrosine kinase (EGFR-TK, PDB ID: 1M17) was selected for prodigiosin’s anticancer potential against skin cancer, aiming to disrupt uncontrolled cell growth [33]. Acidic fibroblast growth factor (FGF-1, PDB ID: 3K1X) docking studied its effect on wound healing processes [34]. Peptide deformylase of *E. faecalis* (PDB ID: 2OS1) was targeted to explore prodigiosin’s antibacterial activity by inhibiting bacterial protein synthesis [35]. The regulator protein of operon involved in the biofilm formation protein of *P. aeruginosa* (PA14_16140, PDB ID: 8Q8O) was chosen to understand prodigiosin’s antibiofilm capacity, aiming to disrupt biofilm formation [36]. Additionally, docking with human peroxiredoxin 5 (PDB ID: 1HD2) assessed prodigiosin’s potential antioxidant activity by interacting with this cellular antioxidant enzyme [37]. The protein preparation module in Schrödinger’s Maestro 13.4 was utilized to preprocess the crystal structures, ensuring they were suitable for docking simulations [38]. The binding pockets of the proteins were identified and characterized using the SiteMap tool [39]. Prodigiosin was prepared as a ligand using the LigPrep module, generating possible ionization states and tautomeric forms [40]. Extra precision (XP) glide docking was employed to predict the stable binding orientations and energies of prodigiosin within the active sites of the selected proteins [41].

### In silico assessment of prodigiosin’s pharmacokinetic and toxicity profile

To gain insights into the pharmacokinetic and toxicity profiles of prodigiosin, in silico computational toxicity predictions were performed using AdmetSAR (admetlab 1.0, http://lmmd.ecust.edu.cn/admetsar1). AdmetSAR is a web-based tool that assesses the absorption, distribution, metabolism, excretion, and toxicity properties of compounds.

### Computational prediction of prodigiosin’s biological activities using the PASS online tool

The investigation of the biological significance of the pigment prodigiosin was conducted through a well-justified computational approach utilizing the PASS (Prediction of Activity Spectra for Substances) online tool (http://www.way2drug.com/PASSOnline/). The PASS online platform is a highly comprehensive computational tool capable of predicting over 4,000 distinct types of biological activities, including pharmacological effects, mechanisms of action, toxicological profiles, interactions with metabolic enzymes and transporters, and influence on gene expression. This broad scope allowed for a thorough exploration of prodigiosin’s potential bioactivity spectrum, providing a detailed understanding of its biological potential [42]. The specific methodology involved inputting prodigiosin’s chemical structure, obtained from the PubChem database (CID: 448530), into the PASS online platform. The prediction parameters were then set to include both the ‘probable activity’ (Pa) and ‘probable inactivity’ (Pi) scores, with a confidence level threshold of 0.5 or higher. The rationale for setting these parameters was to ensure a robust and reliable prediction process. In addition to predicting the potential activities of the compound (Pa), considering the ‘probable inactivity’ (Pi) scores helps to identify the activities that the compound is likely to be inactive against. Moreover, selecting a confidence level threshold of 0.5 or higher guarantees that the predictions have a high level of accuracy and reliability [43]. The comprehensive output data generated by the PASS online tool was carefully reviewed to identify the most relevant bioactivities based on factors such as prediction confidence, novelty, and alignment with the research objectives.

## Results

### Characterization and partial purification of prodigiosin

**Fig. 1:**
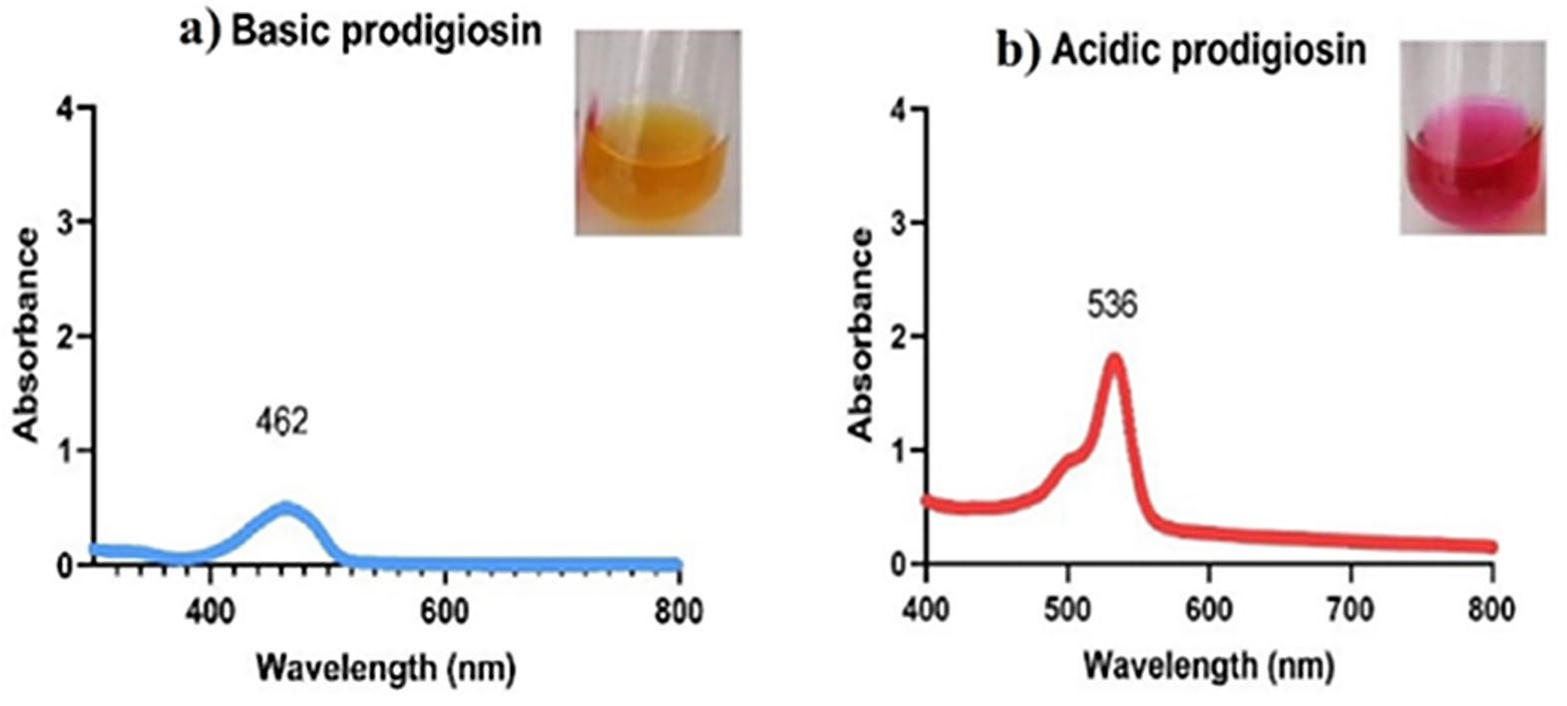
Prodigiosin confirmation and UV-vis spectral analysis at both basic and acidic pH, **(a)** Basic prodigiosin at 462 nm; **(b)** Acidic prodigiosin at 536 nm

**Fig. 2:**
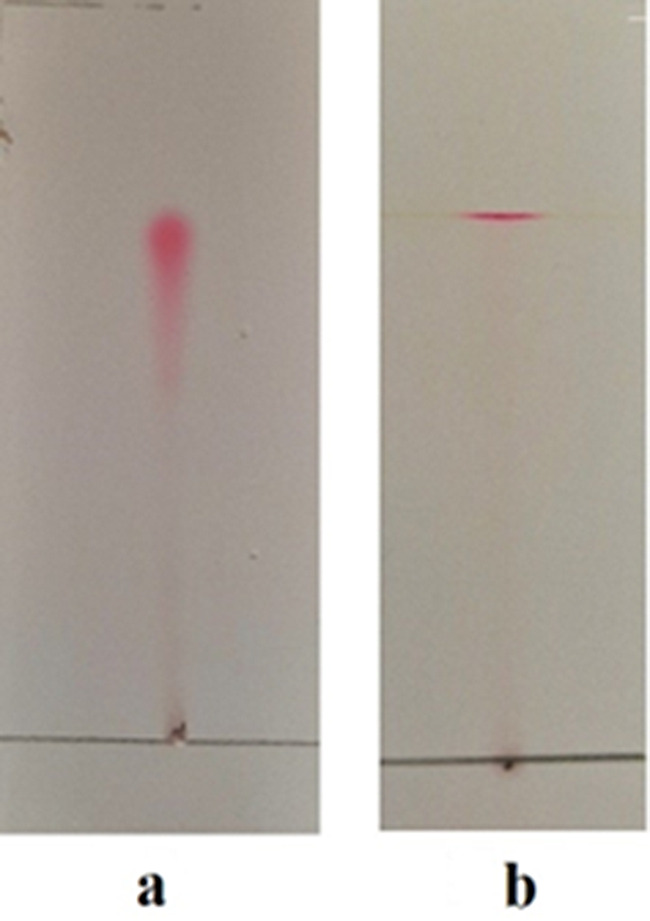
Partial purification of prodigiosin pigment using thin layer chromatography (TLC), (**a**) Before purification, and (**b**) After purification

**Fig. 3:**
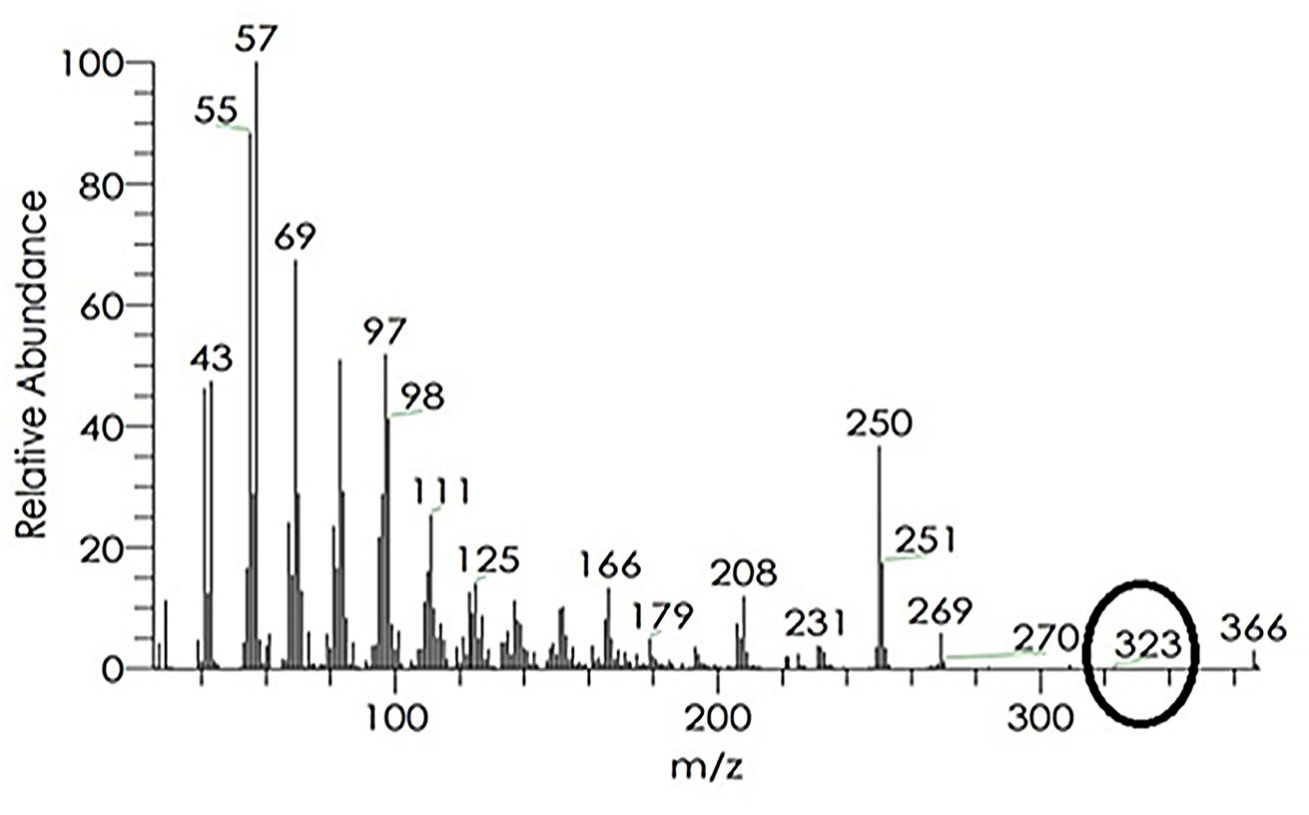
GC-MS analysis of the red fraction (prodigiosin) produced by *S. marcescens* OK482790, and the determined molecular weight was 323 D m/z (black circle)

**Fig. 4:**
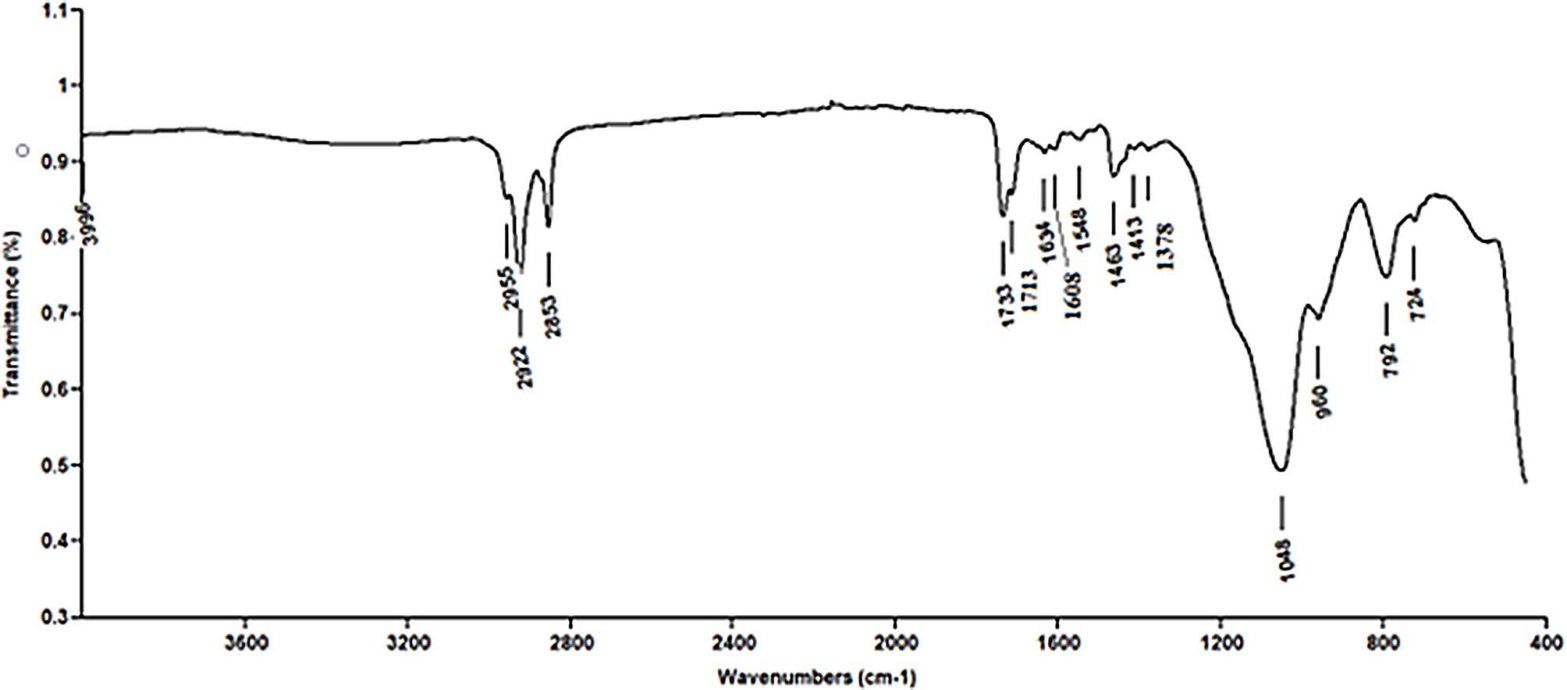
FT-IR spectra of the red prodigiosin pigment. Different functional groups were visible in the spectra at different wavenumbers

At a basic and acidic pH, the extracted red pigment exhibited different colors, yellow and pink, respectively. The absorbance spectra of the basic and acidic prodigiosin pigments in ethanol showed maximum absorbance at 462 nm and 536 nm, respectively (Fig. 1). Following a preliminary identification of the prodigiosin pigment, the pigment was partially purified using TLC; just one pigmented band was found, with an Rf value of 0.92 (Fig. 2). The pigment was scratched, eluted, and characterized by GC/MS. The results demonstrated that the red pigment’s molecular weight was 323 D m/z, showing that the pigment recovered from *S. marcescens* strain OK482790 was prodigiosin pigment (Fig. 3). FT-IR spectra of prodigiosin pigment were detected at different wavenumbers, including 2955 (aromatic C-H stretch), 2922 (H-bonded O-H stretch carboxylic acid or amide-NH group), 2853 (H-bonded O-H stretch or methylene group), 1733 (C = O stretch ester), 1634 (C = O stretch aldehyde or N-H primary amines or pyrrole ring), 1548 (N-H bend secondary amines), 1463 (N-H bend secondary amines), 1378 (C-O group), 1048 (C-N or C-O stretch), 960, 792, and 724 (C-H phenyl) cm^− 1^ (Fig. 4).

### Prodigiosin applications

#### Antibacterial activity

The prodigiosin pigment showed effective antibacterial activity against *Escherichia coli* ATCC 8739, *Listeria monocytogenes* ATCC 7644, *Clostridium perfringens* ATCC 13124, *Staphylococcus aureus* ATCC 6538, *Salmonella typhimurium* ATCC 14028, *Enterococcus faecalis* ATCC 29212, *Shigella sonnei* ATCC 29930, and *Pseudomonas aeruginosa* ATCC 27853, and the activity increased with increasing the concentration, as indicated in (Table 1). The findings also showed that the MIC values for *Enterococcus faecalis* ATCC 29212, *Escherichia coli* ATCC 8739, *Staphylococcus aureus *ATCC 6538, *Clostridium perfringens* ATCC 13124, and *Pseudomonas aeruginosa* ATCC 27853 were 3.9, 62.5, 62.5, 500, and 62.5 µg/mL, respectively.

**Table 1:** MIC values of prodigiosin pigment

Reference bacterial strains		Minimum inhibitory concentration (MIC)		
		µg/mL	C* (1 mg/mL)	CIP* (1 mg/mL)
Gram-positive	*Enterococcus faecalis* ATCC 29212	3.9	0.25	0.125
	*Staphylococcus aureus* ATCC 6538	62.5	0.25	0.5
	*Clostridium perfringens* ATCC 13124	500	0.5	0.5
	*Listeria monocytogenes* ATCC 7644	> 500	0.5	0.25
Gram- negative	*Escherichia coli* ATCC 8739	62.5	0.5	0.25
	*Salmonella typhimurium* ATCC 14028	> 500	0.25	0.25
	*Shigella sonnei* ATCC 29930	> 500	0.25	0.25
	*Pseudomonas aeruginosa* ATCC 27853	62.5	0.25	0.5

*Positive controls (C = chloramphenicol and CIP = ciprofloxacin)

#### Antibiofilm activity

**Fig. 5:**
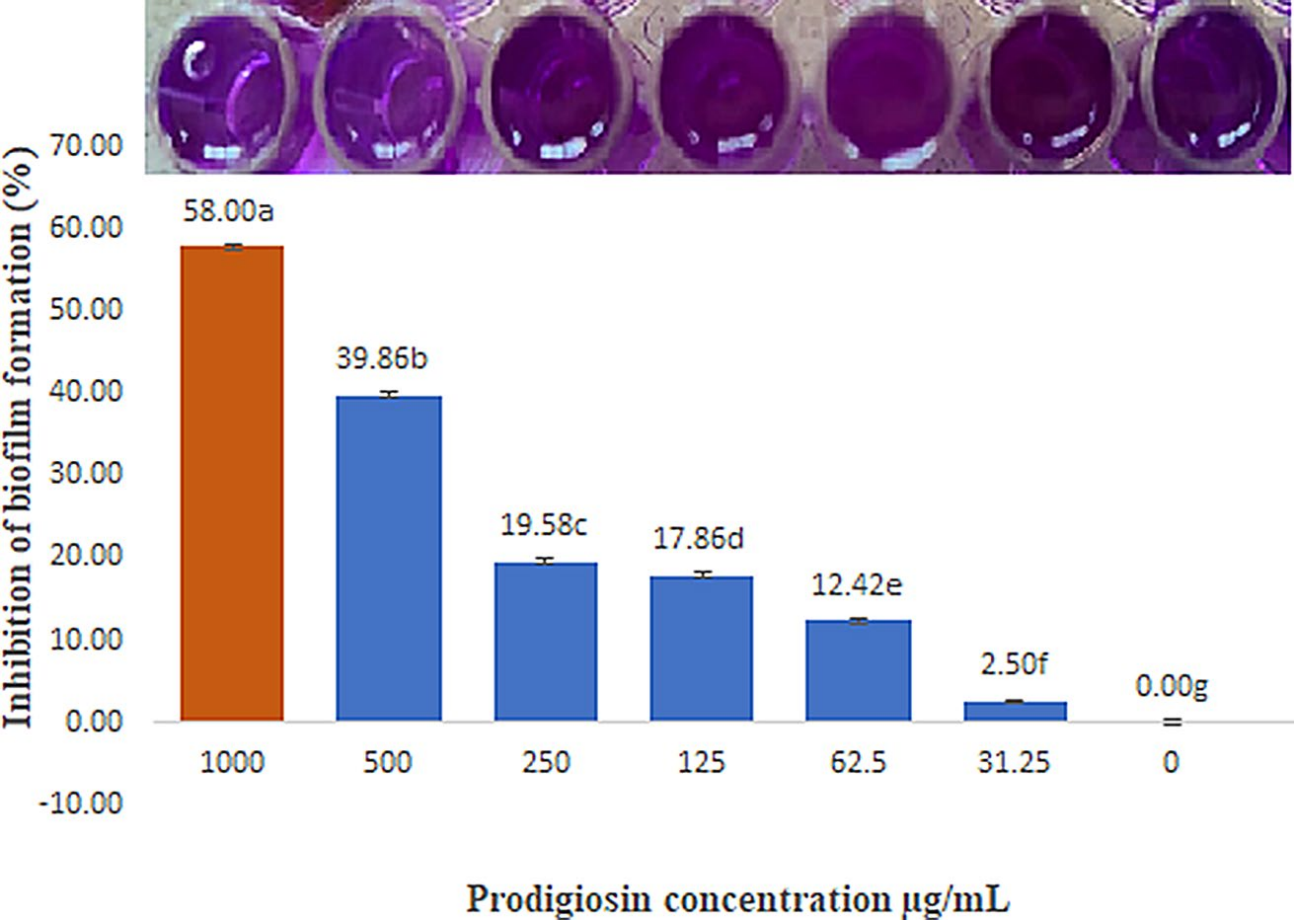
Antibiofilm activity of prodigiosin pigment against biofilm-forming *Pseudomonas aeruginosa* (ATCC 27853). The graph showed that biofilm inhibition was highest (58.00%; orange bar) at a prodigiosin concentration of 1000 µg/mL, followed by inhibition percentages of 39.86, 19.58, 17.86, 12.42, and 2.50% at PG concentrations of 500, 250, 125, 62.5, and 31.25 µg/mL, respectively (blue graphs). The small letters indicate the significance between the different concentrations of prodigiosin according to post-hoc tests (Duncan)

The effect of prodigiosin pigment on the pathogenic *Pseudomonas aeruginosa* ATCC 27,853 biofilm was studied quantitatively using a crystal violet assay with varied concentrations (1000, 500, 250, 125, 62.5, 31.25, and 0 µg/mL). The findings demonstrated that when the pigment concentration increased, the rate of biofilm development significantly decreased, as illustrated in Fig. 5. The inhibition percentage was found to be 58%, 39.86%, 19.58%, 17.86%, 12.42%, and 2.50% at prodigiosin concentrations of 1000, 500, 250, 125, 62.5, and 31.25 µg/mL, respectively.

### Antioxidant activity

**Fig. 6:**
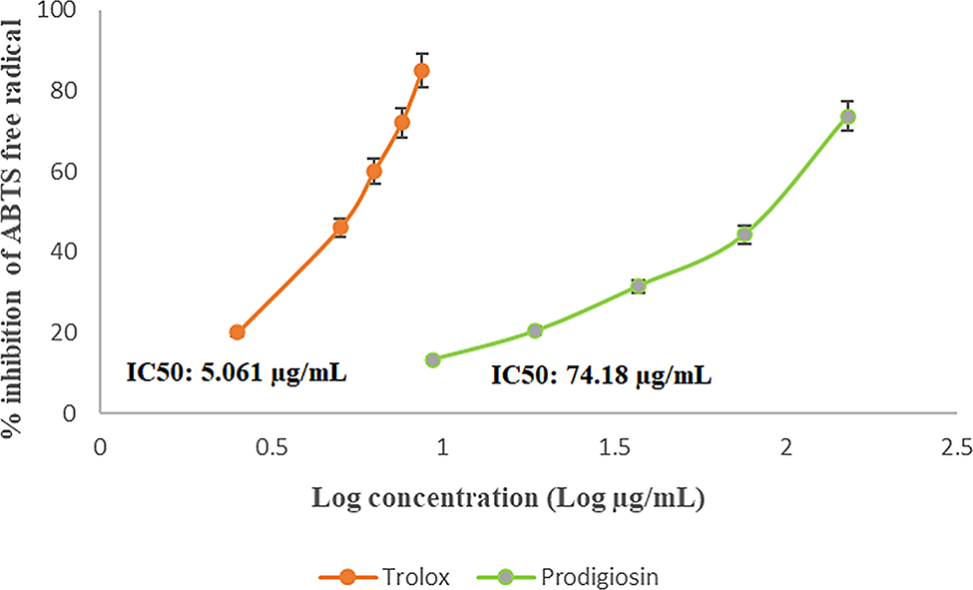
Antioxidant activity of prodigiosin pigment using ABTS assay. Results were indicated as inhibition percentage of ABTS free radical, IC_50_ of prodigiosin (green line) was 74.18 µg/mL, and for positive control (Trolox, orange line) was 5.061 µg/mL. Standard error (± 5%)

The antioxidant capacity of prodigiosin pigment was determined using the ABTS free radical scavenging assay in the presence of Trolox as a standard antioxidant. The results demonstrated that pure PG has considerable ABTS radical scavenging activity based on the dosage, as indicated in Fig. 6. At a concentration of 74.18 ± 23.77 µg/mL, PG demonstrated an IC_50_ of ABTS free radical.

#### Cytotoxicity, anticancer, and wound healing properties of prodigiosin

**Fig. 7:**
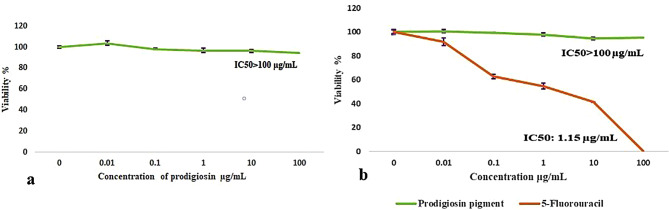
Cytotoxicity and anticancer properties of prodigiosin pigment. **(a)** On normal skin cell line (HSF) with IC_50_ > 100 µg/mL, and **(b)** Its anticancer effect (green line) on human epidermoid skin carcinoma (A-431) using 5-fluorouracil as a positive control (orange line) with IC_50_ > 100 and 1.15 µg/mL, respectively. Standard error bars indicate the standard deviation difference between the groups mean (*n* = 3)

**Fig. 8:**
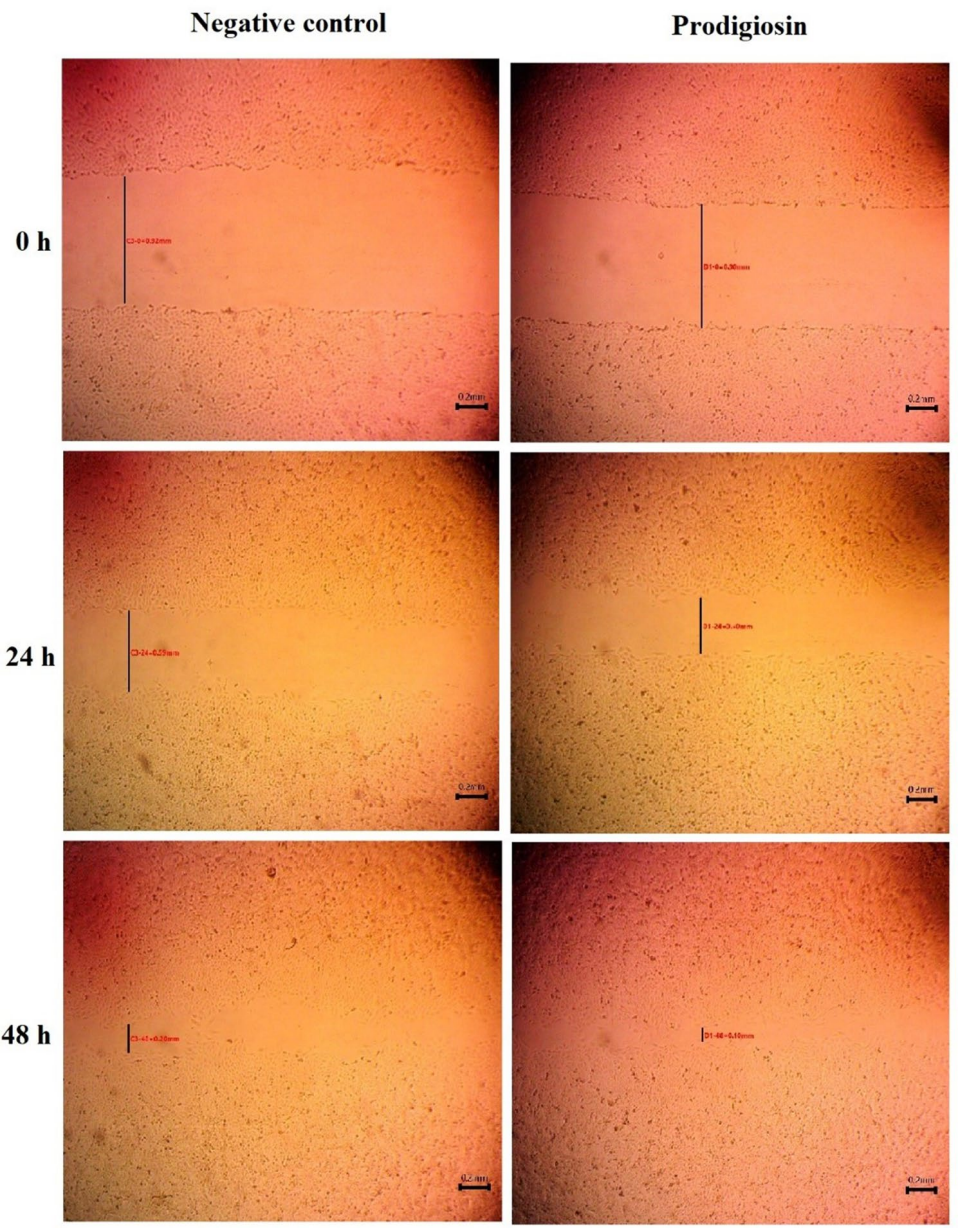
In vitro wound healing properties of prodigiosin pigment using scratch wound assay on human skin fibroblast (HSF) at zero time and after 24, and 48 h incubation with prodigiosin (100 µg/mL). Scale bar = 0.2 mm

Results of cytotoxicity showed that until concentration of 100 µg/mL of prodigiosin, there was no harm effect on the normal skin cell line, and the IC_50_ was higher than 100 µg/mL. Additionally, there was no anticancer effect on human epidermoid skin carcinoma, A-431 (skin/epidermis), until PG concentration of 100 µg/mL, as shown in Fig. 7. The results indicated that PG has a slight impact on wound healing. After 48 h, the scratch width of the PG-treated cells reduced from 0.90 ± 0.0115 mm on the first day (0 h) to 0.13 ± 0.0306 mm (Fig. 8), and the rate of migration increased from zero to 0.9.

#### Molecular docking simulations of prodigiosin against various protein targets

**Fig. 9:**
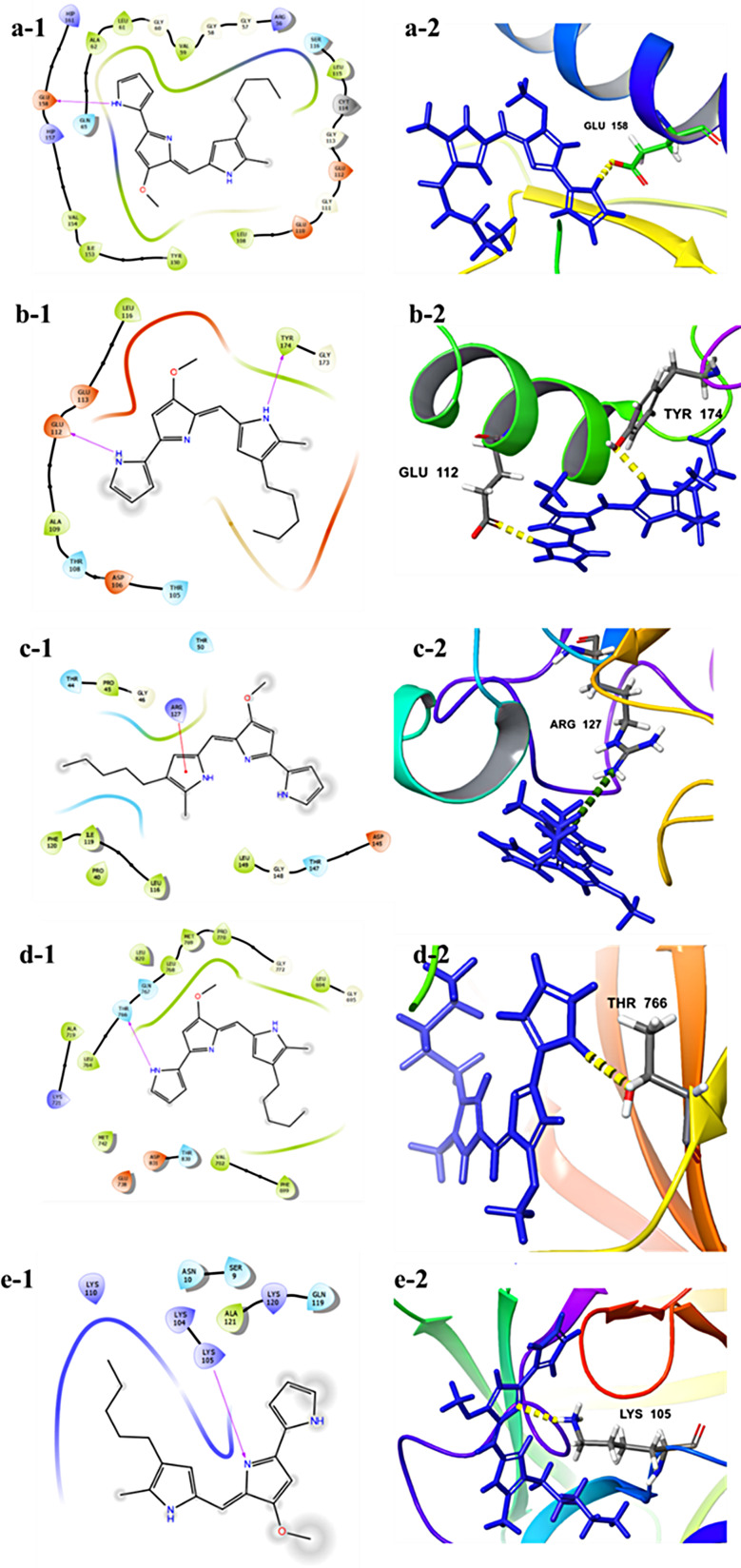
Binding poses of prodigiosin docked to: (**a**) peptide deformylase (*E. faecalis*), (**b**) PA14_16140 (*P. aeruginosa*), (**c**) human peroxiredoxin 5, (**d**) EGFR-TK, and (**e**) FGF-1

Molecular docking was used to investigate the biological activities of prodigiosin, a compound with potential applications in cancer, wound healing, antibacterial, antibiofilm, and antioxidant processes. The results of docking studies showed that prodigiosin binds well to certain proteins, like *E. faecalis’* peptide deformylase, which stops bacteria from synthesizing proteins. It also interfered with EGFR-TK, disrupting uncontrolled cell growth in skin cancer. Prodigiosin’s effects on wound healing were also evident, with a docking score of -2.866 kcal/mol against FGF-1. A docking score of -3.588 kcal/mol against *P. aeruginosa’s* PA14_16140 protein indicated its antibiofilm capacity. Furthermore, its antioxidant activity was indicated by a docking score of -2.423 kcal/mol against human peroxiredoxin 5 (Table 2; Fig. 9).

**Table 2:** Prodigiosin’s binding affinities and interactions with target proteins

Protein	PDB ID	Docking score (kcal/mol)	H-bond	Pi-cation bond
Peptide deformylase (*E. faecalis*)	2OS1	-6.084	GLU 158	N/A
PA14_16140 protein (*P. aeruginosa*)	8Q8O	-3.588	GLU 112 TYR 174	N/A
Human peroxiredoxin 5	1HD2	-2.423	N/A	ARG 127
EGFR-TK	1M17	-5.925	THR 766	N/A
FGF-1	3K1X	-2.866	LYS 105	N/A

N/A; not present

#### In-silico assessment of prodigiosin’s pharmacokinetic and toxicity profiles

Prodigiosin, a drug with potential, has a high human intestinal absorption (HIA+) probability, but its Caco-2 permeability results could suggest limited permeability across the cell monolayer. Its moderate aqueous solubility could pose challenges for formulation and bioavailability. Prodigiosin may accumulate in mitochondria, potentially impacting drug metabolism. It was both a substrate for the CYP450 3A4 enzyme and an inhibitor of the CYP450 1A2 enzyme, potentially leading to drug-drug interactions. Despite its non-toxic status in the AMES test, its acute oral toxicity classification III indicated moderate toxicity (Table 3).

**Table 3:** Predicted ADMET insights for prodigiosin

Variables		Results	Prob
Absorption Human Intestinal Absorption		HIA+	0.9961
Caco-2 Permeability		Caco2-	0.5819
Aqueous solubility [Logs] Distribution		Moderately soluble	-2.7998
Subcellular localization		Mitochondria	0.5160
Metabolism	CYP450 2C9 Substrate	Non-substrate	0.7664
	CYP450 2D6 Substrate	Non-substrate	0.7678
	CYP450 3A4 Substrate	Substrate	0.5309
	CYP450 1A2 Inhibitor	Inhibitor	0.6690
	CYP450 2C9 Inhibitor	Non-inhibitor	0.6347
	CYP450 2D6 Inhibitor	Non-inhibitor	0.7465
	CYP450 2C19 Inhibitor	Non-inhibitor	0.5576
	CYP450 3A4 Inhibitor	Non-inhibitor	0.7057
	CYP Inhibitory Promiscuity	High CYP Inhibitory Promiscuity	0.9045
AMES Toxicity		Non-toxic	0.6432
Carcinogens		Non-carcinogens	0.9040
Biodegradation		Not ready	1.0000
Acute Oral Toxicity		III	0.5881
Carcinogenicity (Three-class)		Non-required	0.5537
Rat Acute Toxicity (LD50, mol/kg)		2.6035	

#### Computational prediction of prodigiosin’s biological activities using the PASS online tool

According to the PASS predictions, prodigiosin had a high probability (Pa > 0.5) of exhibiting various anticancer activities, including an apoptosis agonist (Pa = 0.723), antineoplastic (non-small cell lung cancer, Pa = 0.549; solid tumors, Pa = 0.428; and others), and bcl2 antagonist (Pa = 0.310). Prodigiosin could also boost metabolic and regulatory activities, such as catalase stimulant (Pa = 0.500), calcium regulator (Pa = 0.339), and antianginal (Pa = 0.282). Additionally, prodigiosin displayed cytoprotective and anti-inflammatory properties, including cytoprotectant (Pa = 0.578), immunosuppressant (Pa = 0.493), and antihypertensive (Pa = 0.291) activities (Table 4).

**Table 4:** Biological activities of prodigiosin (PASS online, http://way2drug.com/PassOnline/)

Anticancer activities	Pa%	Metabolic and regulatory activities	Pa%	Cytoprotective and anti-inflammatory activities	Pa%
Apoptosis agonist	0.723	Catalase stimulant	0.500	Cytoprotectant	0.578
Antineoplastic (non-small cell lung cancer)	0.549	Calcium regulator	0.339	Immunosuppressant	0.493
Antineoplastic (solid tumors)	0.428	Inotropic	0.288	Antihypertensive	0.291
Antineoplastic	0.451	Gastric antisecretory	0.173	Anti-Helicobacter pylori	0.232
Antineoplastic (small cell lung cancer)	0.342	Hepatic disorders treatment	0.195	Antiulcerative	0.277
Antineoplastic (lymphoma)	0.228	Liver cirrhosis treatment	0.113	Platelet aggregation inhibitor	0.318
Antineoplastic (glioma)	0.211	Antianginal	0.282	Taurine dehydrogenase inhibitor	0.494
Antineoplastic alkaloid	0.226	Antiischemic	0.191	Gastrin inhibitor	0.511
Antineoplastic (thyroid cancer)	0.173	Age-related macular degeneration treatment	0.201	Thromboxane B2 antagonist	0.478
Antineoplastic (endocrine cancer)	0.161	Erythropoiesis stimulant	0.265	Platelet activating factor antagonist	0.135
Antineoplastic (colorectal cancer)	0.126	Leukopoiesis stimulant	0.281		
Antineoplastic (brain cancer)	0.178	Sulfur reductase inhibitor	0.270		
Antineoplastic (breast cancer)	0.146	Mycothiol-S-conjugate amidase inhibitor	0.256		
Antineoplastic (colon cancer)	0.111	Botulin neurotoxin A light chain inhibitor	0.246		
Bcl2 antagonist	0.310				
Bcl-xL inhibitor	0.107				
RET inhibitor	0.404				

## Discussion

Pigments have become an integral part of our daily lives, with numerous applications in agriculture, textiles, cosmetics, pharmaceuticals, and food [44]. Natural pigments or dyes are a low-cost, biocompatible, and non-toxic option. These pigments are easily accessible through a range of resources, including higher plants (stems, leaves, flowers, and fruits) and microbes [45]. Pailliè-Jiménez et al. reported that many natural pigments, in addition to providing color, are intriguing bioactive compounds that may have beneficial effects on health [44].

This study involved the production and extraction of a natural red pigment from the rhizosphere-isolated strain *Serratia marcescens* (OK482790). The pigment was produced on a solid NA plate, extracted with pure ethanol, subjected to partial purification by thin layer chromatography (TLC), and characterized using several techniques for proving that the isolated pigment was prodigiosin (PG). The pigment production on the solid medium matched the findings of Diken-Gur (2024), who found that the efficient adhesion of bacteria on a solid surface via the quorum sensing (QS) mechanism was responsible for the larger amount of PG produced on the solid medium, and it was proposed that enhanced bacterial communication through this mechanism also caused PG pigment to be produced [16]. The appearance of different colors with different absorbance spectra of the created pigment, yellow in a basic solution at 462 nm and pink in an acidic solution at 536 nm, preliminary defined prodigiosin pigment. This result was nearly agreed with dos Santos et al. (2021), who reported that the red pigment produced by *S. marcescens* UCP 1549 showed maximum absorbance at 535 nm [46]. Furthermore, according to Paul et al. (2022b), the red hue of prodigiosin is due to its absorption maxima at 535 nm, which were given by acidified ethanol [47]. In addition, Paul et al. (2022a) found that, depending on the solvent pH, prodigiosin solubilized in methanol had absorption maxima determined via a UV-visible spectrophotometer ranging from 460 to 540 nm [19]. For instance, at pH 7.0, prodigiosin maintains its red hue and absorption maximum at 533 nm, but at pH 2.0 (pink color) and 9.0 (orange color), its absorption maximum is at 540 nm and 468 nm, respectively [48]. An alkaline pH may cause the N atoms in pyrrole rings to be deprotonated, which could explain why the absorption maxima move to a higher pH [49]. Recently, Lu et al. reported that according to spectrophotometric examination, PG can appear orange-yellow in alkaline settings (with a broad curve centered at 470 nm) or red (with a strong peak at 535 nm) in acidic media [15]. This work, like other investigations [50, 51], utilized the red form of prodigiosin pigment in neutral pH to determine its chemical and medicinal properties. All experiments were conducted on the naturally extracted prodigiosin (red) without adding any acid or base.

The GC/MS data has proved that the red pigment’s molecular mass extracted from *Serratia marcescens* (OK482790) was 323 D m/z. This outcome was matched with the findings of Vitale et al., who suggested that the two significant peaks with m/z values of 322.1914 and 324.2071 corresponded to prodigiosin and cycloprodigiosin, respectively [52]. Also, Lapenda et al. results showed that the obtained pigment from *Serratia marcescens* strain UFPEDA 398 had a molecular weight of 323 D m/z and a maximum absorption at 534 nm, which corresponded to prodigiosin [53]. Additionally, data from Venil et al. stated that, on GC-MS, the pigment’s molecular mass was 323 D m/z, which matches that of prodigiosin (C_20_H_25_N_3O_) [54]. Thin-layer chromatography using a chloroform: methanol (9:1 v/v) solvent solution yielded just one band that has an R_f_ of 0.92. Previous literature Someya et al. (2004) stated that prodigiosin is the red pigment with an R_f_ value between 0.90 and 0.95 [55]. Similar studies were given by Bhagwat & Padalia, which found that R_f_ values for prodigiosin extracts dissolved in methanol and DMSO were 0.87 and 0.89, respectively [3]. Moreover, dos Santos et al. highlighted that the R_f_ value of the produced red pigment equals 0.92 [46]. The prodigiosin pigment’s functional groups include pyrrole, amide, methylene, methyl, and alkene [56]. In this investigation, the FT-IR spectra of prodigiosin pigment recorded distinct wavenumbers that corresponded to different functional groups. The results were nearly aligned with those reported by Venil et al., who found that the pigment’s FTIR absorption spectra were primarily characterized by two distinct bands at 2925.46 and 1402.75 cm^− 1^ for aromatic CH and aromatic C = C, respectively [54]. Furthermore, the results were in accordance with those obtained by Paul et al. (2022b), who recorded peaks at frequencies 2923 cm^− 1^, 2854 cm^− 1^, 1734 cm^− 1^, 1629 cm^− 1^, 1546 cm^− 1^, 1465 cm^− 1^, 1229 cm^− 1^, 1069 cm^− 1^, 1026 cm^− 1^, and 720 cm^− 1^ that corresponded to H bonded O-H stretch carboxylic acid, H bonded O-H stretch, C = O stretch ester, C = O stretch aldehyde or N-H primary amines, N-H bend secondary amines, N-H bend secondary amines, C-O stretch ether/ester, C-O stretch ether/ester, C-N stretch or C-O stretch, and C-H phenyl, respectively [47]. Earlier findings by Sumathi et al. (2014) showed that the presence of the C-O group in prodigiosin caused a peak to appear at 1379 cm^− 1^, and the carbon-carbon double bond caused peaks around 1293 cm^− 1^ and 718 cm^− 1^ [57].

Prodigiosin has many biological activities, such as the ability to destroy cancer cells, lethal to bacteria and fungi, effective against malaria, protozoa, and other parasites, suppresses the immune system, slows cell proliferation, and fights tumors [58, 59]. Cyclic compounds, like prodigiosin, exhibit distinct antibacterial action than linear ones. Its structure is a tripyrrole ring. The metabolic stability and cell permeability of cyclic compounds are both higher than those of their linear counterparts [60]. In this finding, different medicinal bioactivities of PG as antibacterial, antibiofilm, antioxidant, anticancer, and wound healing agents were performed. For the in vitro antioxidant, cytotoxicity, and anticancer bioactivities, the starting concentrations were 150, 100, and 100 µg/mL, respectively, and the selection of these low concentrations was based on toxicity considerations, ensuring prodigiosin was safe and effective without significantly harming untargeted cells or molecules. Based on the cytotoxicity results, the concentration (100 µg/mL) used in the wound healing test was chosen, and the reason behind this is that prodigiosin pigment was not harmful up to 100 µg/mL (IC_50_ > 100 µg/mL). However, in the antibacterial and antibiofilm assays, the initial concentrations were increased to ensure a positive result against the used bacterial strains. Further research would be employed to optimize the concentration dependency for each assay. Both Gram-positive and Gram-negative bacteria were effectively inhibited by PG’s antibacterial action [60]. The MIC values of PG isolated from OK482790 for *Enterococcus faecalis* ATCC 29212, *Escherichia coli* ATCC 8739, *Staphylococcus aureus* ATCC 6538, *Clostridium perfringens* ATCC 13124, and *Pseudomonas aeruginosa* ATCC 27853 were 3.9, 62.5, 62.5, 500, and 62.5 µg/mL, respectively. Following the same line, Ibrahim et al. (2023) found that PG was effective against *Bacillus cereus*, *S. aureus*, *E. coli*, and *P. aeruginosa* with MIC values of 1.0 ± 0.15, 5.0 ± 0.25, 16.5 ± 0.87, and 16.5 ± 0.92 µg/mL, respectively [61]. Results from Yip et al. (2021) noticed that PG effectively reduced the growth of *S. aureus*, *E. faecalis*, and *E. coli* bacteria, with MIC values of ≥ 10 µg/µL, but the values were not evaluated for both *P. aeruginosa* and *Salmonella Typhimurium* [60]. According to Jardak et al. (2022), prodigiosin showed a strong antibacterial action selectively against two types of bacteria: Gram-positive *S. aureus* with a minimum inhibitory concentration (MIC) ranging from 78 to 156 µg/mL and Gram-negative *E. coli* with a MIC value ranging from 39 to 78 µg/mL [62]. PG exerts its antibacterial effect by targeting DNA, resulting in DNA cleavage, cell cycle disruption, pH changes, phototoxicity, enhanced hydrophobic stress, and the generation of oxygen radicals [60]. Previous studies by Ravindran et al. (2020) examined prodigiosin-membrane interactions through molecular dynamics research and suggested that the membrane immediately receives solvent-derived prodigiosin, either individually or in small clusters. After that, when molecules enter, they form clusters at the water-membrane interface, where pyrrole rings interact with water and lipid head groups. Hydrogen-hydrophobic interactions maintain this orientation and then prodigiosin molecules modify membrane lipid structure and limit solvent accessibility [63].

Biofilms are produced by pathogenic bacteria in response to harsh conditions and to safeguard themselves from antibiotics and other antimicrobial agents [60]. Scientists therefore seek to develop safe antibiofilm agents in order to combat bacterial resistance. Biofilm formation by *Pseudomonas aeruginosa* ATCC 27,853 was tested in the presence and absence of PG from *S. marcescens* OK482790 using a crystal violet assay. In this typical technique, the liquid medium and planktonic cells are drained, leaving only attached biofilm, and crystal violet dye is applied for a period of time. This dye is retained by the biofilm matrix, and by adding ethanol as a decolorizing agent, the dye is resolubilized, and the amount of biofilm in the sample could be estimated quantitatively using spectrophotometer [64, 65]. The findings showed inhibition of *P. aeruginosa*-formed biofilm that was treated with PG, with a percentage reaching 58% at a prodigiosin concentration of 1000 µg/mL. Similarly, results from Jardak et al. showed inhibition of biofilm formation by *Staphylococcus epidermidis* S61 when mixed with different doses of prodigiosin (30 µg/mL to 10 mg/mL) [66]. Matched results were obtained by Yip et al. (2021), who reported that biofilm formation was significantly lower in *E. faecalis* and *Salmonella typhimurium* that had been treated with prodigiosin compared to the untreated bacteria [60]. In a prior study, prodigiosin from *Serratia marcescens* blocked *Pseudomonas aeruginosa* from forming biofilm at concentrations of 500 µM and 100 µM when copper was complexed with it. In addition, the relation between prodigiosin and limiting the development of biofilm via making redox active metabolites was reported [67]. PG induces strong RNA and dsDNA cleavage, but they don’t have a big effect on protein by creating H_2_O_2_ and hydroxyl radicals. Prodigiosin, or the prodigiosin/copper (II) complex, greatly changed the hydrophobicity of *P. aeruginosa* cell surfaces and the integrity of biofilms by inhibiting interaction forces responsible for aggregation and bacterial adhesion [67]. In this study, at a concentration of 74.18 ± 23.77 µg/mL, PG demonstrated an IC_50_ of ABTS free radical. The ABTS assay was chosen precisely in our work to avoid inaccurate results that may occur as a result of the red color of prodigiosin pigment. The test result depended on the change of ABTS^+^ blue-green color to colorless, and the used wavelength for absorbance was 734 nm. In order to avoid color interference, Arnao (2000) advised choosing a wavelength that is outside of the visible spectrum and stated that in the case of the ABTS^+^ test, the problem was less serious since the chromogen exhibits absorbance peaks at 730 and 842 nm [68]. Additionally, results were represented as log values to reduce the dispersion of the data set and to help in getting a linear response. Since the response data is always a nonlinear number, the behavior of nonlinear numbers needs to be described by nonlinear mathematics and plotted on a nonlinear logarithmic scale [69]. This outcome was consistent with Sudhakar et al., who reported that at a concentration of 500 µg/mL, PG demonstrated an ABTS scavenging activity of 71 ± 1.3% and noted also that the bioactive PG derived from *S. marcescens* showed a strong antioxidant potential that varied with dose [21]. The antioxidant activity could be attributed to the conjugated double bonds and ring pyrrole structures of PG [61]. This study found that PG from OK482790 up to 100 µg/mL had no hazardous effect on normal skin, so we got excited to apply it as a wound healing agent, and the results revealed that red pigment PG has a moderate effect on repairing wounded cells. Hacene (2020) noticed that when prodigiosin was applied topically, the skin healed completely in 6 days [70]. Furthermore, anticancer results were unclear, with IC_50_ exceeding 100 µg/mL. This outcome was not in agreement with results obtained by Lapenda et al., which recorded that prodigiosin produced by *S. marcescens* UFPEDA 398 had cytotoxic activity against cell lines isolated from solid tumors (NCIH-292, Hep-2, and MCF-7) and leukemia (HL-60) with an IC_50_ of 3.6 µg/mL for all studied tumor cell lines, except the MCF-7 line (IC_50_ 5.5 µg/mL) and mentioned that the cytotoxic effect was related to inducing cell death by DNA fragmentation [53]. A previous study by Anwar et al. reviewed that a red pigment prodigiosin generated by *Serratia marcescens* has inherent anticancer properties, demonstrating remarkable antitumor actions in diverse malignancies (e.g., breast, stomach) with low or no negative effects on normal cells [71]. Moreover, Jardak et al. noticed that the newly isolated *Serratia* sp. C6LB produced prodigiosin with cytotoxic activity against breast cancer lines MCF-7 and MDA-MB231, with IC_50_ values of 16 µg/mL and 6.7 µg/mL, respectively [66]. The healing of wounds is a key part of restoring the skin’s protection function. Many different types of cells work together to make this process happen. It can be broken down into five stages: hemostasis, inflammation, proliferation, migration, and reorganization. Scientists often use in vitro models to study how wounds heal, looking at things like cell migration, proliferation, protein production, wound closure, and the interactions between cells and the matrix [72]. The in vitro scratch wound technique can test cell migration, an important step in wound healing [73]. At this stage, keratinocytes and fibroblasts multiply and move to the wound site, where fibroblasts make granulation tissues from extracellular matrix (ECM). Angiogenesis is also crucial at this stage because it brings blood to newly made tissue [74]. The results of this study indicated that PG at a concentration of 100 µg/mL has a minor effect on wound healing compared with the negative control. Since cell migration causes the wound width to decrease [30], the R_m_ increased from zero at the beginning of the experiment to 0.9 after 48 h. In contrast, a recent study by Hamzah and Awayid (2023) involved isolating *Serratia marcescens* from Iraqi central hospitals, such as those in the City of Medicine as well as other major hospitals. It demonstrated the ability to produce virulence factors at various levels based on virulence genes in its genome, which increased pathogenicity and infection spread [75]. This indicates that the variation in the isolation source reflects the variability of the medical importance of *Serratia marcescens*.

The molecular docking experiments conducted in this study offer important new insights into the possible mechanisms of action that underlie prodigiosin’s various bioactivities. We were able to clarify the compound’s potential modes of interaction and binding affinities by focusing on particular proteins linked to different therapeutic domains, which provided insight into its multifunctional characteristics. Prodigiosin’s docking with the epidermal growth factor receptor tyrosine kinase (EGFR-TK, PDB ID: 1M17) suggested that it has the potential to disrupt the uncontrolled cell growth signaling mediated by this receptor. Since EGFR-TK is a key regulator of cell proliferation, its aberrant activation is often linked to the development of various cancers, including skin cancer [76]. The binding of prodigiosin to the active site of EGFR-TK indicated that the compound may be able to interfere with the receptor’s kinase activity, thereby inhibiting the downstream signaling cascades that promote uncontrolled cell division and tumor growth, and this result validated prodigiosin’s anticancer properties, especially against skin cancer cells. Additionally, prodigiosin’s ability to affect wound healing processes was revealed by the results of its docking with acidic fibroblast growth factor (FGF-1, PDB ID: 3K1X). FGF-1 is an essential growth factor that is involved in angiogenesis, proliferation, and cell migration at different stages of wound healing [52]. Prodigiosin’s steady binding to FGF-1 may alter how the growth factor interacts with other signaling molecules or its specific receptors, which may have an impact on the cellular reactions involved in wound healing. This implied that prodigiosin’s ability to control FGF-1 activity would improve or hasten the healing of wounds. Prodigiosin’s antibacterial mechanism of action was clarified by docking it with the bacterial peptide deformylase enzyme from *E. faecalis* (PDB ID: 2OS1). An important stage in the maturation of proteins is the removal of the N-formyl group from freshly generated bacterial proteins, which is accomplished by the enzyme peptide deformylase [77]. The binding of prodigiosin to this enzyme could inhibit its function, thereby disrupting bacterial protein synthesis and ultimately leading to cell death; this provided a logical explanation for the observed antibacterial activity of prodigiosin against *E. faecalis*. Prodigiosin docking with the PA14_16140 protein from *P. aeruginosa* (PDB ID: 8Q8O) provided insight into the compound’s antibiofilm potential. This protein is believed to play a role in the formation and maintenance of bacterial biofilms, which are complex multicellular structures that provide protection and enhanced survival for the bacterial community [78]. The binding of prodigiosin to this protein could disrupt its function, thereby compromising the ability of *P. aeruginosa* to form and maintain its biofilm matrix. This result validated prodigiosin’s antibiofilm activity against this opportunistic pathogen. Lastly, the docking of prodigiosin with human peroxiredoxin 5 (PDB ID: 1HD2) provided a mechanistic understanding of the compound’s antioxidant properties. Peroxiredoxin 5 is a cellular antioxidant enzyme that helps neutralize various reactive oxygen species, thereby protecting the cell from oxidative stress [79]. Prodigiosin’s binding to peroxiredoxin 5 could increase or modify the enzyme’s capacity to scavenge free radicals and reactive oxygen species, which could explain why PG was known to have antioxidant properties. This result implied that prodigiosin might function as a natural antioxidant, with possible uses in a range of conditions linked to oxidative stress. Acharya et al. identified prodigiosin as a potent quorum-sensing inhibitor and anti-biofilm agent against *Acinetobacter baumannii* through in silico docking analysis and experimental validation [80]. Additionally, Sudhakar et al. reported the production, optimization, and characterization of prodigiosin from *Serratia marcescens* strain CSK, which exhibited strong antioxidant, antibacterial, and anticancer activities, including its potential binding to the caspase-3 protein [21].

The ADMET predictions for prodigiosin offer valuable insights into its potential pharmacokinetic and safety profiles. The results showed a high human intestinal absorption probability, suggesting favorable absorption characteristics. However, the Caco-2 permeability results suggested that there was limited permeability across the Caco-2 cell monolayer. Prodigiosin’s moderate aqueous solubility could pose challenges for formulation and bioavailability. Its subcellular localization suggested it may accumulate in mitochondria, affecting its mechanism of action and potential therapeutic targeting. Prodigiosin was predicted to be a substrate for the CYP450 3A4 enzyme and an inhibitor of the CYP450 1A2 enzyme, potentially leading to drug-drug interactions. Its toxicity predictions suggested it is non-toxic in the AMES test and not considered a carcinogen. However, the acute oral toxicity classification of III indicated a moderate level of toxicity. Anwar et al. reported that prodigiosin, a secondary metabolite red pigment produced by *S. marcescens*, exhibited favorable ADMET properties as evidenced by its drug-likeness profile [81].

The comprehensive PASS predictions highlighted the diverse biological activities of prodigiosin, which span anticancer, metabolic, regulatory, cytoprotective, and anti-inflammatory domains. The high probability scores for various anticancer activities, such as apoptosis agonists and antineoplastic effects against different types of cancer, suggested that prodigiosin could be a promising lead compound for further investigation in the field of oncology. The metabolic and regulatory activities, including catalase stimulation, calcium regulation, and antianginal effects, indicated prodigiosin’s potential for managing metabolic disorders and cardiovascular conditions. Furthermore, the cytoprotective and anti-inflammatory properties, such as immunosuppression and antihypertensive effects, suggested that prodigiosin may have applications in inflammatory and autoimmune-related diseases. Koyun et al. reported that the prodigiosin pigment produced by the *S. marcescens* MB703 strain showed promising bioactivities, including anticancer and neuroprotective effects, as predicted by the PASS online tool [56].

However, the process of producing prodigiosin took a significant amount of time due to the need to harvest the bacterial cells with pigments from the surface of the nutrient agar plates. Therefore, a fermenter is needed. Moreover, large volumes of solvents, such as ethanol and chloroform, were used for the extraction and purification processes, and several TLC sheets were employed during the purification step. But, the results of our lab-produced PG as antibacterial, antibiofilm, antioxidant, anticancer, and wound healing agents were suggestive for a promising use of PG from OK482790 as alternative medicine since it is considered a natural product and has valuable benefits as inherently safer and gentler on the body compared to synthetic pharmaceuticals, and this can provide a sense of security and comfort. Using prodigiosin is avoiding the side effects that could result from the active drugs found in the current medications. In addition, using prodigiosin in wound healing, for example, will help the wounds heal and prevent them from being contaminated with bacteria, thus accelerating the healing process. Future research will be involved in studying the anti-inflammatory properties, in vivo studies on animals to confirm PG effectiveness, clinical research studies will be considered on a small number of healthy volunteers to study the effectiveness of prodigiosin and determine the appropriate dose, then on a larger number of patients to study its effectiveness and compare it to currently available treatments, and as a result of previously mentioned features of our produced PG and its red color, further future studies will be done to apply prodigiosin pigment from OK482790 in the preparation of different skin cosmetics. In addition, the future research of prodigiosin’s bioactivities could be direct to fields other than medicine, such as using prodigiosin in the bioremediation process as a plant growth-promoting substance, production of nanoparticles from prodigiosin, and conjugation of prodigiosin with nanoparticles.

## Conclusion

The current study relied on a red pigment extracted from the rhizosphere bacterium *Serratia marcescens* (OK482790), characterized, and identified as prodigiosin (PG). In vitro and in silico studies have shown PG’s biological activity as an antibacterial, antibiofilm, antioxidant, and wound healing agent. The results indicated that PG is an effective natural alternative to many therapeutic drugs.

## Data Availability

All data supporting the findings of this study are available within the manuscript. Regarding the accession number of *Serratia marcescens* is available on https://www.ncbi.nlm.nih.gov/nuccore/OK482790

## Abbreviations

PG: Prodigiosin
R_f_: Retardation factor
ABTS 2,2’: Azino-bis (3-ethylbenzothiazoline-6-sulfonic acid)
PASS: Prediction of activity spectra for substances
